# Drinking-Water Herbicide Exposure in Indiana and Prevalence of Small-for-Gestational-Age and Preterm Delivery

**DOI:** 10.1289/ehp.0900784

**Published:** 2009-07-31

**Authors:** Hugo Ochoa-Acuña, Jane Frankenberger, Leighanne Hahn, Cristina Carbajo

**Affiliations:** 1 Epidemiology and Public Health Section, Comparative Pathobiology; 2 Agricultural and Biological Engineering; 3 Office of the Indiana State Chemist Office and; 4 Earth and Atmospheric Sciences, Purdue University, West Lafayette, Indiana, USA

**Keywords:** atrazine, birth weight, epidemiology, herbicides, preterm birth

## Abstract

**Background:**

Atrazine and other corn herbicides are routinely detected in drinking water. Two studies on potential association of atrazine with small-for-gestational-age (SGA) and preterm birth prevalence found inconsistent results. Moreover, these studies did not control for individual-level potential confounders.

**Objectives:**

Our retrospective cohort study evaluated whether atrazine in drinking water is associated with increased prevalence of SGA and preterm birth.

**Methods:**

We developed atrazine concentration time series for 19 water systems in Indiana from 1993 to 2007 and selected all births (*n* = 24,154) based on geocoded mother’s residences. Log-binomial models were used to estimate prevalence ratios (PRs) for SGA and preterm delivery in relation to atrazine concentrations during various periods of the pregnancy. Models controlled for maternal demographic characteristics, prenatal care and reproductive history, and behavioral risk factors (smoking, drinking, drug use).

**Results:**

Atrazine in drinking water during the third trimester and the entire pregnancy was associated with a significant increase in the prevalence of SGA. Atrazine in drinking water > 0.1 μg/L during the third trimester resulted in a 17–19% increase in the prevalence of SGA compared with the control group (< 0.1 μg/L). Mean atrazine concentrations over the entire pregnancy > 0.644 μg/L were associated with higher SGA prevalence than in the control group (adjusted PR = 1.14; 95% confidence interval, 1.03–1.24). No significant association was found for preterm delivery.

**Conclusions:**

We found that atrazine, and perhaps other co-occurring herbicides in drinking water, is associated with an increased prevalence of SGA, but not preterm delivery.

There has been a sustained increase in preterm delivery (< 37 weeks gestation) and small-for-gestational-age (SGA; defined as birth weight below the 10th percentile for a given sex and gestational week) prevalence in the United States in the last few decades. This is a cause of concern because these conditions are associated with increased risks of infant morbidity and mortality, as well as health problems in later life (Behrman and Butler 2007). Numerous studies have attempted to determine whether environmental exposures, such as consumption of contaminated drinking water during pregnancy, play a role in the observed increase in preterm delivery and SGA prevalence. Most of these studies have focused on water disinfection by-products (e.g., Hoffman et al. 2008a, 2008b; Joyce et al. 2008; Lewis et al. 2007; Yang 2004; Yang et al. 2007). In contrast, only a handful have focused on other water contaminants, such as pesticides (Bukowski et al. 2001; Munger et al. 1997; Villanueva et al. 2005).

In many areas of the U.S. Midwest, the landscape is dominated by agricultural crops routinely treated with various pesticides. In Indiana, approximately 21–27% of the total area is planted each year with corn [U.S. Department of Agriculture (USDA) 2007], and more than 85% of this area is treated with the herbicide atrazine (6-chloro-N2-ethyl-N4-isopropyl-1,3,5-triazine-2,4-diamine) (CropLife Foundation 2006). As a consequence, many community water systems (CWSs) distribute finished drinking water containing atrazine (Hua et al. 2006; McMillin and Means 1996). At least during spring and summer, concentrations of this herbicide are significant, and exceedances of the federal maximum contaminant level (MCL) of 3.0 μg/L (U.S. Environmental Protection Agency (EPA) 2003) are common.

To date, there have been only two studies relating concentrations of atrazine in drinking water and adverse pregnancy outcomes. Munger et al. (1997) reported that the percentage of SGA babies was higher in Iowa communities with a mean annual atrazine concentration of > 2.2 μg/L compared with communities with < 0.8 μg/L atrazine. However, this study did not control for the fact that atrazine concentrations vary widely throughout the year within the same water source. Villanueva et al. (2005) found no association between atrazine concentrations in drinking water in French communities and prevalence of SGA, prematurity, or low birth weight. However, this lack of association might have been attributable to differences in concentrations between unexposed and exposed groups that were rather small (< 0.029 μg/L in the control group and > 0.036 μg/L in the high exposure group).

Our objective was to test the potential association between atrazine concentrations in drinking water during different periods of the pregnancy and prevalence of preterm delivery and SGA. We hypothesized that preterm delivery may be related to exposures occurring during early pregnancy, because developmental abnormalities, which have been shown to increase the risk of preterm delivery, occur during the first weeks of pregnancy (Dolan et al. 2007). We also hypothesized that pre-term delivery may alternatively be caused by a direct effect on the mother, prompting early labor, which is likely to be triggered by events occurring shortly before delivery. We further hypothesized that effects on intrauterine growth may be related to exposures occurring during the last trimester or during the entire pregnancy. We based this study on a detailed atrazine concentrations data set developed over the years as part of several monitoring efforts and the spatial demarcation of CWS service area boundaries. These data were used to estimate the drinking-water exposure to atrazine of mothers living within these service areas.

## Materials and Methods

### Exposure concentrations

We selected CWSs that obtained their water from a surface water source, that served urban areas, and for which there was certainty about the boundaries of the CWS service area. We selected these CWSs to allow unequivocal assignment of birth records to CWSs using the geographic coordinates of each mother’s residence. For all but two CWSs, boundaries were ascertained from the 2000 census of populated areas that were part of a CWS, based on information from the Indiana Department of Environmental Management (IDEM) [U.S. Department of Commerce (USDOC) 2008]. In the case of Bloomington CWS, service area boundaries were ascertained based on digital map layers of the surface water district boundary provided by the CWS. For one CWS (Stucker Fork), boundaries were delineated based on scanning and digitizing a paper map of water pipelines and a hand-drawn, 0.5-mile buffer around them. After all CWS boundaries were completed, the information was compiled into one ArcGIS shape-file (version 9.2; ESRI, Redlands, CA).

Pesticide concentrations in drinking water are not regularly collected by any agency, except for the quarterly samples required of all public water systems under the Safe Drinking Water Act (1974). This temporal resolution is not sufficient for resolving short-term exposures or even reconstructing cumulative exposure during the entire pregnancy. We had access to three additional data sets of atrazine concentrations in finished drinking water in Indiana, each of which contained records from biweekly sampling for periods of several months to several years. These data were collected as part of the Acetochlor Registration Partnership (Hackett et al. 2005), Novartis Atrazine Public Water System Voluntary Monitoring (Tierney et al. 1999), and the Atrazine and Simazine Re-registration Program (U.S. EPA 2006). Combined with the data from IDEM (Safe Drinking Water Act samples), atrazine concentrations in finished drinking water could be reconstructed for surface drinking water systems between 1993 and 2007. The Acetochlor Registration Partnership took place from 1995 to 2001. Acetochlor, alachlor, atrazine, and metolachlor were included in this study (Hackett et al. 2005). The Novartis Atrazine Public Water System Voluntary Monitoring Program measured atrazine and simazine concentrations from 1993 to 1996 (Tierney et al. 1999). Finally, Syngenta began the Atrazine Monitoring Program in 2003, which focused on atrazine concentrations (U.S. EPA 2006). All these monitoring efforts sampled atrazine in drinking water every 7–14 days during the spring/summer and less often during the rest of the year. From these data sets, we selected sampling periods when sampling occurred at least biweekly during the spring and summer and where at least two samples were collected and analyzed during fall and winter. In the case of Bloomington, we obviated this requirement because this CWS has had atrazine concentrations consistently below the detection limit and therefore was not the object of intense sampling.

### Pregnancy outcomes

Infant and mother information was retrieved from the Indiana Birth Records Database. This database contains > 100 variables describing demographic characteristics of the mother, her health, health attitudes, pregnancy outcomes, procedures performed before and during delivery, and the geographic coordinates of the mother’s domicile for all babies delivered in Indiana. The Institutional Review Board of Purdue University and the Indiana State Department of Health approved the study protocol using the selected records. Birth locations were matched to specific CWSs by using the latitude and longitude coordinates of the mother’s domicile location at the time of birth. To ensure balanced representation of all periods throughout the annual cycle, and to properly reconstruct concentrations for the entire pregnancy, we selected for each CWS pregnancies occurring when continuous atrazine concentration records were available for whole years, and for which atrazine concentrations were available for the entire pregnancy. The result of this selection was that although births were selected from all the CWSs included in the study, the number of births from each CWS varied greatly, with most records coming from Ft. Wayne. We determined gestation length based on the date of the last menses or on the gestation length in weeks reported in the registry. We excluded records from multiple pregnancies or terminated by cesarean section or by induction of labor. We also excluded records with gestation lengths < 22 weeks or > 44 weeks, given that births < 22 weeks are rarely viable and that births are usually induced before the 44th week of gestation. Finally, we also excluded birth weights < 200 g and > 6,000 g, because these weights are likely the result of misreporting. Births were considered preterm if they occurred before the 37th week of pregnancy. Babies were considered SGA if their weight at birth was below the 10th percentile of birth weights for their sex at the given gestation week, based on data from more than 6 million singleton births occurring during 1999–2000 in the United States (Oken et al. 2003).

### Data analysis

We created continuous atrazine concentration time series for each CWS by linear interpolation between sampling dates using PROC EXPAND (version 9.1; SAS Institute Inc., Cary, NC, USA). Then, depending on the birth date and length of gestation, we calculated the average atrazine concentrations for the first and last month of pregnancy and for the entire pregnancy. We aimed at determining the association between atrazine concentrations during the first and last month of pregnancy and preterm delivery and pregnancy duration. For SGA and term birth weight, we focused on the average atrazine concentration during the last trimester for term births and the entire pregnancy for all births. For each pregnancy period studied, we divided birth records into low, medium, and high groups according to the concentration of atrazine in drinking water. We used the 25th and 75th percentiles as cutoffs. Measures of association were also estimated using atrazine concentrations as continuous measures.

**Figure 1:**
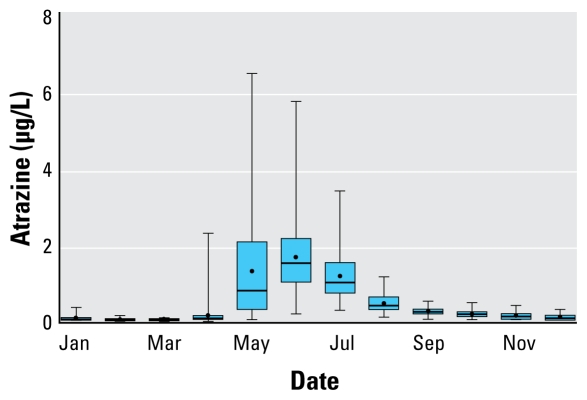
Seasonal pattern of atrazine concentrations in finished drinking water in Ft. Wayne, Indiana, USA. Dots indicate mean, lines median, boxes the interquartile range (25th–75th percentiles), and error bars the 5th–95th percentiles.

Association between atrazine concentrations and the response variables was studied using a two-step process. First, we ran log-binomial models using PROC GENMOD (SAS Institute Inc.) to identify potential confounders to be included in the final models. Confounders considered included infant’s sex and mother’s ethnicity, age, marital status, education, prenatal care, previous reproductive history, health status during pregnancy, and smoking, drinking, and drug use. In addition to these variables, we also included the quarter of the year in which conception occurred to account for any seasonal difference in preterm births and SGA and the known seasonal trends observed for atrazine concentrations in drinking water (Figure 1). Confounders were dropped if they did not cause a > 1.0% change in the prevalence ratio (PR) of the atrazine level variable compared with the fully parameterized model and if their level of significance was < 0.1. PRs and their respective 95% confidence intervals (CIs) were then estimated by fitting the final log-binomial models using PROC GENMOD ESTIMATE option. Duration of pregnancy in weeks and term birth weights (≥ 37 weeks) were also fitted to linear models that included atrazine concentrations and confounders as explanatory variables. In the case of term birth weights, we also included gestation length in weeks and its quadratic term in the models to account for growth leveling off toward the end of pregnancy (Ritz and Yu 1999).

## Results

Drinking-water atrazine concentration data are summarized in Table 1 for each CWS. Atrazine tended to decrease to below detection levels in the winter months in all CWSs (data not shown). Although median annual concentrations were similar among several CWSs (Evansville, Ft. Wayne, Richmond, Jasper), maximum values, as well as the number of days atrazine concentrations exceeded 0.2 and 0.5 μg/L, varied greatly among CWSs. A total of 24,154 birth records were linked to atrazine and included in this study. The number of births varied among CWSs because of different population size and number of years with adequate atrazine data. Most birth records (67.8%) were from one CWS, Ft. Wayne.

### Preterm delivery and pregnancy duration

Maternal characteristics for the preterm and SGA prevalence studies are presented in Table 2. Overall, prevalence of preterm delivery was 7.36% (95% CI, 7.18–7.54). Preterm delivery was more prevalent for male, compared with female, babies and for babies born to unmarried mothers (Table 2). Preterm delivery prevalence decreased with maternal age until 26–28 years and then increased. African-American and Native-American mothers had higher preterm delivery prevalence than white (including Hispanic) or Asian-American mothers; Hispanic mothers had lower prevalence than non-Hispanics. We also observed that prevalence varied with parity, with the lowest rate for the second birth. Mothers with a history of a previous infant death or of fetal death had preterm prevalence almost twice as high as the overall value. Preterm delivery prevalence decreased with increasing level of education attained by the mother and mothers commencing prenatal care during the first month of pregnancy had lower prevalence than the overall value. Mothers who self-reported smoking, drinking, and drug use during pregnancy had higher rates of preterm delivery than mothers not reporting these behaviors. Mothers suffering from a concurrent disease during pregnancy had higher prevalence than healthy mothers.

Results from our analysis comparing groups of mothers exposed to different levels of atrazine in drinking water showed that prevalence of preterm delivery was not significantly associated with atrazine during the first or last months of pregnancy when adjusting for potential confounders (Table 3).

The overall average duration of pregnancy was 38.8 ± 2.11 weeks. Our analysis on the prevalence of preterm delivery found that when atrazine was included in the model as a continuous variable, it was not significantly associated with preterm delivery after controlling for known confounders (adjusted PR = 1.07; 95% CI, 0.99–1.15). In addition, atrazine concentration during the last month of pregnancy was not significantly associated with prevalence of preterm delivery when considered as a continuous variable (PR = 0.99; 95% CI, 0.92–1.06).

### SGA and term birth weight

The prevalence of SGA was 13.13% (95% CI, 12.95–13.31) (Table 2). Male babies had a higher prevalence of SGA than female babies, and unmarried mothers had a higher prevalence of SGA than married ones. Prevalence of SGA decreased with maternal age and was higher in black and Asian-American mothers compared with white mothers. We also found that the second child from a mother had the lowest prevalence of SGA and that mothers with a previous fetal death or live-born infant dying after birth had a higher prevalence of SGA. Prevalence of SGA also decreased with maternal education, and mothers who started prenatal care during the first month of pregnancy had lower SGA prevalence. Prevalence of SGA increased with self-reported use of tobacco, alcohol, and drug during pregnancy, and mothers who were ill during pregnancy also had elevated SGA prevalence.

When using mean atrazine concentration during the third trimester of pregnancy as the exposure metric for term babies, we found that prevalence of SGA increased from 11.0% in the reference group (i.e., < 25th percentile, or 0.103 μg/L atrazine) to 14.3% for medium and 13.1% for the high atrazine group. Prevalence of SGA in the medium and high atrazine groups were increased by 19 and 17%, respectively (Table 3). We also found that the (log-transformed) mean atrazine concentration during the third trimester was associated with an increase in the prevalence of SGA (adjusted PR = 1.12; 95% CI, 1.07–1.17). Term (≥ 37 weeks) birth weight in the study population was 3,338 ± 457 g (mean ± SE). Atrazine concentrations during the third trimester were also significantly associated with a decrease in term birth weight (*p* = 0.0011) after controlling for potential confounders using linear models that also included gestational duration.

In the case of exposures quantified as the mean atrazine concentration over the entire pregnancy, prevalence of SGA increased from 12.0% in the reference group (i.e., < 25th percentile, or 0.179 μg/L atrazine for the entire pregnancy) to 13.3% for medium and 13.9% for the high atrazine group. Prevalence of SGA in the high atrazine group was 14% higher than in the control group (adjusted PR = 1.14; 95% CI, 1.03–1.24). We also found that the prevalence of SGA was significantly associated with atrazine when this was included as a continuous variable in the models (adjusted PR = 1.15; 95% CI, 1.04–1.28). A 1.0-μg/L increase in mean atrazine concentration in drinking water for the entire pregnancy was associated with an average increase of 15% in the prevalence of SGA. We found that mean atrazine concentrations in drinking water over the entire pregnancy were also significantly associated with a decrease in term birth weight. We found that birth weight decreased by 34.06 ± 10.7 g (mean ± SE) for every additional microgram per liter of atrazine in drinking water (*p* = 0.0015). We also found that when restricting our analysis to Ft. Wayne, which included most of the observations of the study, the analysis resulted in a similar trend, with the highest exposure group (> 0.742 μg/L) having an 11% increase in the prevalence of SGA (adjusted PR = 1.11; 95% CI, 1.00–1.24) compared with the lowest exposure group (< 0.320 μg/L).

## Discussion

The results of this study suggest that prenatal exposure to atrazine in drinking water is associated with reduced birth weight, but not preterm delivery. Only two previous studies have looked at the potential association between atrazine in drinking water and adverse pregnancy outcomes (Munger et al. 1997; Villanueva et al. 2005). The Munger et al. (1997) study was based on comparing prevalence of SGA of 13 communities served by a CWS with high levels of atrazine with mothers living in communities of different size and from the same counties. This study reported that atrazine and other herbicide concentrations were significant predictors of community prevalence of SGA but not preterm delivery. However, this ecologic study did not attempt to control for seasonal changes in atrazine concentrations and lacked proper control of individual-level potential confounders. The Villanueva et al. (2005) study was based on assigning birth records to different exposure levels estimated as the overall mean of atrazine concentrations measured over an 8-year period, irrespective of when each pregnancy occurred. This study did not detect an association between atrazine in drinking water and prevalence of preterm delivery, low birth weight, or SGA. Villanueva at al. (2005) hypothesized that this lack of association may have been due to the fact that exposure concentrations were relatively low, with cutoff values for the medium and high exposure groups of 0.029 and 0.036 μg/L, respectively (Villanueva et al. 2005). In the case of Munger et al. (1997), exposed and unexposed communities had a mean atrazine concentration of 2.2 and 0.7 μg/L, respectively.

The observed association may not represent an effect due to exposure solely to atrazine, but rather also to other co-occurring chemicals. We had access to finished drinking-water concentration data for other herbicides collected from a small subset of the samples used in this study. The herbicides acetochlor, alachlor, and metolachlor were all correlated with atrazine (correlation coefficients, *r* = 0.65, 0.54, and 0.84, respectively). However, these herbicides were detected much less frequently than atrazine and at significantly lower concentrations. Hackett et al. (2005) reported that the occurrence of annualized mean concentrations of acetochlor, alachlor, and metolachlor exceeding 0.1 μg/L in finished drinking water was 8.1, 2.9, and 2.2%, respectively. In contrast, atrazine concentrations exceeded 0.1 μg/L 74.4% of the time. The extent to which this study demonstrates an association between adverse pregnancy outcomes and atrazine or, alternatively, herbicides in general, cannot be ascertained at this time.

A significant source of uncertainty in studies that use drinking-water concentration as the exposure metric is that the level of municipal, unfiltered water consumption of individual subjects is unknown. Pregnant women may choose to use bottled water over tap water, at least for some proportion of their water needs. Forssen et al. (2007) found that of 2,300 women, 28% used bottled water and 19% used filtered tap water. This is an increase from a 2001 study of 114 women, which showed that 25% drank bottled or filtered water (Zender et al. 2001). Women who work outside of the home or travel may also be exposed to alternative water supplies. More than 30% of water consumption for pregnant women occurred outside of the home (Kaur et al. 2004). We have no information for our study population regarding the level of use of tap water or whether the same level of use exists for different CWSs or times of the year.

A potential source of misclassification in spatial epidemiologic studies results from the address at birth not representing the address at conception, either through incorrect data or if the subject moved between conception and delivery. Many studies that evaluated birth events used the address of the mother at the time of birth, as found on the birth certificate. Using the address given at the time of delivery may introduce misclassification to studies on the association between health effects and maternal environmental exposure during pregnancy (Fell et al. 2004; Shaw et al. 1999). Mobility rates for pregnant women have varied among studies, from 12% (Fell et al. 2004), 20% (Khoury et al. 1988), and 25% (Shaw et al. 1999) to 32% (Zender et al. 2001). Although these data suggest a potential concern for relating drinking water to health outcomes, 62–69% of the women who moved did so within the same county or water municipality in these study populations (Fell et al. 2004; Shaw et al. 1999). Unfortunately, no estimates of maternal residential mobility during pregnancy exist for the state of Indiana.

A major strength of the present study is the ability to control for several potential confounding factors. The effects and maternal confounder data used in this study were retrieved from the Indiana State Department of Health Birth Certificates database. This database contains information entered by hand from hospital records, so there is a chance of incorrect data entry. However, a study (Zollinger et al. 2006) conducted to assess the accuracy and completeness of this database compared with hospital records found that important descriptive and outcome data variables were reliable, whereas data for infrequent events were not. There was good agreement in the case of birth weight and prematurity as well as for demographic data, and moderate agreement among behavioral risk factors (Zollinger et al. 2006). However, we were not able to determine whether bias was introduced by incorrect determination of gestational length or untruthful self-reporting of behavioral risk factors, such as smoking and alcohol and drug use by the mother.

Our data set included a wide array of CWSs that had different temporal profiles of atrazine concentrations in drinking water. Although all CWSs where atrazine was routinely detected tended to have very low concentrations during the winter months, the magnitude and duration of concentration peaks varied among CWSs. A potential problem with these data is that other, uncontrolled differences among CWSs may have contributed to the observed association between atrazine and adverse pregnancy outcomes. We evaluated this possibility by restricting our analysis to Ft. Wayne, which included most of the observations of the study. This reduced the difference in atrazine concentrations among exposure groups. Nevertheless, the analysis of Ft. Wayne resulted in a similar trend, with the highest exposure group (> 0.742 μg/L) for the entire pregnancy having an 11% increase in the prevalence of SGA (adjusted PR = 1.11; 95% CI, 1.00–1.24) compared with the lowest exposure group (< 0.320 μg/L).

Unlike most other studies relating concentrations of chemicals in drinking water and adverse health effects, we had detailed atrazine concentrations times-series data. Although some exposure misclassification might have occurred because of extrapolation of atrazine concentrations, their impact is likely small. We evaluated exposure based on mean values for the first and last months of pregnancy and for the entire pregnancy. Given that concentration data for periods of the year when atrazine concentrations increase over background were collected every 7–14 days, it is unlikely that interpolation of these data resulted in significant bias. On the other hand, interpolation of the data to produce continuous atrazine concentration time series allowed for proper averaging of atrazine concentrations for the entire pregnancy.

The limited experimental data seem to provide biologic plausibility to the observed association between atrazine in drinking water and adverse pregnancy outcomes. Atrazine exposure *in utero* caused decreased body weight of rat male offspring at day 4 postpartum (Rayner et al. 2007). *In vitro* tests using murine preimplantation embryos showed that atrazine at concentrations derived from the oral reference dose significantly increased the level of apoptosis and decreased the proportion of embryos developing to blastocyst, both alone and in mixture with metolachlor, 2,4-D (2,4-dichlorophenoxyacetic acid), and ammonium nitrate (Greenlee et al. 2004). However, the extent to which these data obtained from animal models are predictive of adverse effects in humans, especially of preterm delivery and SGA, is unknown.

In conclusion, in this study we found a significant association between atrazine concentration in drinking water and prevalence of SGA. However, it is not clear at present whether this association represents a true cause–effect relationship, as other co-occurring chemicals in drinking water were significantly correlated with atrazine. The large number of people in the Midwest who are seasonally exposed to these chemicals through consumption and use of drinking water indicates that these associations be explored further.

## Figures and Tables

**Table 1:** Summary of atrazine data for Indiana CWSs included in the study.

CWS	No. of years^a^	No. of births	Atrazine concentrations^b^		Mean (± SD) no. of days atrazine		Preterm prevalence (95% CI)^c^	SGA prevalence (95% CI)^c^
			Annual median	Annual maximum	> 0.2 μg/L	> 0.5 μg/L		
Batesville	7	233	0.996 ± 0.278	1.988 ± 0.774	18 ± 13	15 ± 10	7.30 (6.85–7.74)	6.01 (5.64–6.38)
Bedford	4	255	0.101 ± 0.124	5.282 ± 7.643	7 ± 11	4 ± 7	8.24 (7.76–8.71)	8.24 (7.76–8.71)
Bloomington	9	1,849	0.058 ± 0.170	0.096 ± 0.207	11 ± 30	0	6.60 (6.45–6.74)	8.49 (8.31–8.67)
Dubois	3	134	0.080 ± 0.026	0.110 ± 0.039	0	0	9.70 (8.94–10.5)	9.70 (8.94–10.5)
Evansville	4	2,296	0.158 ± 0.073	0.987 ± 0.265	63 ± 60	26 ± 28	6.79 (6.66–6.93)	14.1 (13.8–14.3)
Ft. Wayne	9	16,393	0.194 ± 0.090	4.290 ± 2.108	265 ± 218	129 ± 100	7.58 (7.52–7.63)	13.7 (13.6–13.8)
Indiana American Northwest	1	692	0.001	0.001	0	0	3.90 (3.76–4.04)	12.9 (12.4–13.3)
Indiana American Kokomo	1	59	0.305	0.856	106 ± 149	13 ± 18	11.9 (10.5–13.2)	5.08 (4.46–5.71)
Indiana American Richmond	2	324	0.261 ± 0.071	0.490 ± 0.014	0	0	12.3 (11.7–12.9)	12.3 (11.7–12.9)
Jasper	8	280	0.214 ± 0.098	2.623 ± 2.882	12 ± 12	5 ± 6	7.14 (6.75–7.54)	8.21 (7.76–8.66)
Logansport	2	216	0.120 ± 0.087	5.936 ± 8.069	17 ± 28	6 ± 10	6.48 (6.07–6.89)	13.9 (13.1–14.7)
Michigan City	3	848	0.011 ± 0.008	0.028 ± 0.017	0	0	7.19 (6.96–7.42)	17.0 (16.5–17.5)
Mitchell	3	64	0.066 ± 0.061	1.045 ± 1.700	2 ± 4	2 ± 4	9.38 (8.31–10.4)	9.38 (8.31–10.4)
Mt. Vernon	3	90	0.087 ± 0.032	0.965 ± 0.636	4 ± 3	2 ± 3	[Table-fn tfn1-ehp-117-1619]—	6.67 (6.01–7.32)
Northwest Indiana	1	165	0.001	0.001	0	0	5.45 (5.05–5.86)	14.5 (13.6–15.5)
Oakland City	3	22	0.154 ± 0.063	0.193 ± 0.086	0	0	9.09 (7.33–10.9)	[Table-fn tfn1-ehp-117-1619]—
Salem	4	100	0.294 ± 0.338	0.604 ± 0.506	6 ± 8	0	5.00 (4.53–5.48)	12.0 (10.9–13.1)
Scottsburg	4	65	0.435 ± 0.535	0.647 ± 0.549	4 ± 6	2 ± 3	9.23 (8.19–10.3)	10.8 (9.58–12.0)
Stucker Fork	4	69	0.094 ± 0.083	3.379 ± 4.096	3 ± 3	0	8.70 (7.74–9.65)	18.8 (17.0–20.7)

—, no data.

a Includes only years with complete atrazine time series.

b Values represent the mean of annual median and maximum concentrations.

c Prevalence values in percentage (per 100).

**Table 2:** Total number of births and cases and prevalence of preterm delivery and SGA births by characteristics of the mother.

	Preterm delivery			SGA		
Variable	No. of births or mean ± SD	No. of cases or mean ± SD	Prevalence (95% CI)	No. of births or mean ± SD	No. of cases or mean ± SD	Prevalence (95% CI)
All records	24,154	1,777	7.36 (7.18–7.54)	24,154	3,172	13.1 (12.9–13.3)
Mean gestational age (weeks)	39.23 ± 1.12	33.96 ± 3.13		38.85 ± 1.99	38.79 ± 1.64	
Mean birth weight (g)	3,338 ± 457	2,367 ± 731		3,367 ± 501	2,604 ± 309	
SGA (< 10th percentile)	3,172	230	7.25 (7.13–7.37)			
Preterm (< 37 weeks)				1,777	230	12.9 (12.7–13.2)
Sex of infant						
Female	11,984	799	6.67 (6.61–6.72)	11,984	1,476	12.3 (12.2–12.4)
Male	12,170	978	8.04 (7.97–8.10)	12,170	1,696	13.9 (13.8–14.0)
Baby conceived during						
First quarter of year	5,456	414	7.59 (7.49–7.68)	5,456	720	13.2 (13.0–13.3)
Second quarter of year	5,754	430	7.47 (7.38–7.56)	5,754	801	13.9 (13.8–14.1)
Third quarter of year	6,346	453	7.14 (7.06–7.22)	6,346	797	12.6 (12.4–12.7)
Fourth quarter of year	6,598	480	7.27 (7.19–7.36)	6,598	854	12.9 (12.8–13.1)
Marital status						
Married	12,710	794	6.25 (6.20–6.30)	12,710	1,165	9.17 (9.09–9.24)
Unmarried	11,443	983	8.59 (8.52–8.66)	11,443	2,006	17.5 (17.4–17.7)
Maternal age (years)						
< 22	7,287	634	8.70 (8.61–8.79)	7,287	1,247	17.1 (16.9–17.2)
22–25	6,226	439	7.05 (6.97–7.13)	6,226	833	13.4 (13.2–13.5)
26–28	3,874	238	6.14 (6.05–6.24)	3,874	418	10.8 (10.6–10.9)
29–32	3,855	260	6.74 (6.64–6.85)	3,855	377	9.78 (9.64–9.92)
> 32	2,912	206	7.07 (6.95–7.20)	2,912	297	10.2 (10.0–10.4)
Maternal race						
African American	4,599	451	9.81 (9.68–9.94)	4,599	974	21.2 (20.9–21.4)
Asian/Pacific islander	200	14	7.00 (6.54–7.46)	200	30	15.0 (14.1–15.9)
Native American	42	7	16.7 (14.5–18.8)	42	1	2.38 (2.02–2.74)
White^a^	18,770	1,268	6.76 (6.71–6.80)	18,770	2,087	11.1 (11.0–11.2)
Other	454	28	6.17 (5.90–6.44)	454	70	15.4 (14.8–16.0)
Ethnicity						
Hispanic origin	1,969	126	6.40 (6.26–6.53)	1,969	250	12.7 (12.4–12.9)
Non-Hispanic	22,185	1,651	7.44 (7.40–7.49)	22,185	2,922	13.2 (13.1–13.2)
Parity						
First birth	9,510	793	8.34 (8.26–8.42)	9,510	1,458	15.3 (15.2–15.5)
Second birth	7,567	478	6.32 (6.25–6.38)	7,567	841	11.1 (11.0–11.2)
Third or subsequent birth	7,047	502	7.12 (7.04–7.20)	7,047	868	12.3 (12.2–12.4)
Previous infant deaths	392	50	12.8 (12.2–13.3)	392	51	13.0 (12.4–13.6)
Previous fetal deaths (≥1)	31	4	12.9 (10.9–14.9)	31	9	29.0 (25.3–32.7)
Maternal education						
< High school	6,379	562	8.81 (8.71–8.91)	6,379	1,197	18.8 (18.6–19.0)
High school	8,510	638	7.50 (7.42–7.57)	8,510	1,202	14.1 (14.0–14.3)
Some college	5,077	360	7.09 (7.00–7.18)	5,077	512	10.1 (10.0–10.2)
4-year college	4,188	217	5.18 (5.11–5.26)	4,188	261	6.23 (6.14–6.32)
Month prenatal care began						
1st	3,553	216	6.08 (5.98–6.18)	3,553	366	10.3 (10.1–10.5)
2nd	8,272	593	7.17 (7.1–7.24)	8,272	1,004	12.1 (12.0–12.2)
3rd	6,352	447	7.04 (6.96–7.12)	6,352	805	12.7 (12.5–12.8)
> 3rd	5,233	398	7.61 (7.51–7.70)	5,233	847	16.2 (16.0–16.4)
Not a WIC participant	12,583	846	6.72 (6.67–6.78)	12,583	1,252	9.90 (9.87–10.0)
WIC participant	11,569	930	8.04 (7.97–8.11)	11,569	1,920	16.6 (16.5–16.7)
Maternal smoking						
Did not smoke	18,662	1,286	6.89 (6.84–6.94)	18,662	2,018	10.8 (10.7–10.9)
Smoked 1–5 cigarettes/day	1,336	110	8.23 (8.03–8.44)	1,336	265	19.8 (19.4–20.3)
Smoked 6–9 cigarettes/day	789	75	9.51 (9.20–9.81)	789	152	19.3 (18.7–19.8)
Smoked 10–19 cigarettes/day	2,044	180	8.81 (8.63–8.98)	2,044	414	20.2 (19.9–20.6)
Smoked 20–29 cigarettes/day	1,061	95	8.95 (8.70–9.20)	1,061	260	24.5 (23.9–25.1)
Smoked > 29 cigarettes/day	191	22	11.5 (10.8–12.3)	191	47	24.6 (23.3–25.9)
Mother did not drink during pregnancy	23,837	1,742	7.31 (7.26–7.35)	23,837	3,097	13.0 (12.9–13.1)
Mother drank during pregnancy	207	21	10.1 (9.51–10.8)	207	51	24.6 (23.3–25.9)
Mother did not used drugs during pregnancy	23,771	1,710	7.19 (7.15–7.24)	23,771	3,065	12.9 (12.8–13.0)
Mother used drugs during pregnancy	309	59	19.1 (18.2–20.0)	309	96	31.1 (29.8–32.3)
Mother was not ill during pregnancy	19,574	1,366	6.98 (6.93–7.03)	19,574	2,531	12.9 (12.8–13.0)
Mother was ill during pregnancy	4,580	411	8.97 (8.85–9.09)	4,580	641	14.0 (13.8–14.2)

WIC, Special Supplemental Nutrition Program for Women, Infants, and Children.

a Includes Caucasians of Hispanic origin.

**Table 3:** Prevalence of preterm delivery and SGA in relation to mean level of atrazine in drinking water (μg/L) and adjusted PRs (95% CI) for comparisons between medium (≥ 25th, ≤ 75th percentiles), and high (> 75th percentile) and the control exposure group (< 25th percentile).

Response	Atrazine exposure group (μg/L)^a^	Within-group percentiles			No. of births	Gestation length (weeks ± SD) or birth weight (g ± SD)	No. of preterm/SFA cases	Preterm/SGA prevalence (CI)	Preterm/SGA adjusted PR (CI)^b^
		25th	50th	75th					
Preterm delivery									
First month	< 0.057	0.001	0.020	0.050	4,995	38.9 ± 2.02	358	7.17 (7.07–7.26)	
	0.057 to 0.435	0.100	0.165	0.256	10,072	38.8 ± 1.95	736	7.31 (7.24–7.37)	0.98 (0.87–1.11)
	> 0.435	0.655	1.121	1.781	5,034	38.8 ± 1.96	402	7.99 (7.88–8.09)	1.07 (0.93–1.22)
Last month	< 0.057	0.001	0.037	0.050	5,407	38.9 ± 2.00	393	7.27 (7.18–7.36)	
	0.057–0.507	0.100	0.180	0.281	10,889	38.8 ± 1.96	818	7.51 (7.45–7.58)	1.04 (0.93–1.18)
	> 0.507	0.768	1.227	1.884	5,443	38.8 ± 1.95	409	7.51 (7.42–7.61)	0.87 (0.72–1.04)

SGA									
Third trimester^c^	< 0.103	0.001	0.045	0.050	4,363	3,309 ± 528	479	11.0 (10.8–11.1)	
	0.103–0.835	0.117	0.210	0.326	8,747	3,268 ± 526	1,251	14.3 (14.2–14.4)	1.19 (1.08–1.32)
	> 0.835	0.872	1.116	1.482	4,373	3,276 ± 514	575	13.1 (13.0–13.3)	1.17 (1.03–1.34)
Entire pregnancy	< 0.179	0.001	0.047	0.107	6,038	3,284 ± 568	723	12.0 (11.8–12.1)	
	0.179–0.644	0.277	0.363	0.491	12,078	3,273 ± 534	1,609	13.3 (13.2–13.4)	1.06 (0.98–1.15)
	> 0.644	0.740	0.822	0.950	6,038	3,237 ± 543	840	13.9 (13.8–14.1)	1.14 (1.03–1.24)

a Values listed correspond to < 25th, 25th–75th, and > 75th percentiles.

b Adjusted for mother’s ethnicity, level of education, month prenatal care began, smoking status, and quarter of the year in which baby was conceived.

c Excludes records from preterm deliveries (< 37 weeks’ gestation).
